# Reconstruction of native cellular microanatomy in a novel bioengineered full thickness human nasal mucosal construct

**DOI:** 10.1111/joa.70130

**Published:** 2026-03-16

**Authors:** Steven Bradbury, Bethany Dickson, Nicola Curtis, David Bunton, Kirsty Goncalves, Stefan Przyborski

**Affiliations:** ^1^ Department of Biosciences Durham University Durham UK; ^2^ AstraZeneca Liverpool UK; ^3^ REPROCELL Europe Limited Glasgow UK

**Keywords:** bioengineered tissue equivalent, ciliated pseudostratified epithelium, electron microscopy, human respiratory nasal mucosa, microanatomy, submucosal extracellular matrix

## Abstract

Bioengineered tissues offer vital platforms capable of fundamental research, screening interventions and reducing the use of animals in scientific research. However, their predictive accuracy is dependent upon how closely their structure and function resemble their native counterpart. In this study, we present a novel in vitro engineered construct representative of the human nasal mucosa, consisting of both stromal and epithelial compartments. Communication between the stroma and the overlying epithelium is an essential factor involved in tissue development and homeostasis yet is often lacking in the majority of published tissue constructs. Described is the construction of a pseudostratified epithelium consisting of an organised heterogeneous cell population consistent with the respiratory region of the nasal mucosa that forms on a stromal foundation populated with tissue‐specific fibroblasts and endogenous extracellular matrix. In addition, for the first time, we provide an extensive ultrastructural analysis of a bioengineered nasal tissue using both scanning and transmission electron microscopic techniques. This in‐depth characterisation revealed microanatomical hallmarks consistent with the native tissue, including motile cilia, mucin secretions, intercellular junctions, dynamic basement membrane, microvilli and glycocalyx. Given each of these features play pivotal physiological roles in the specialised functions of the respiratory epithelium, their presence in vitro lends itself to tissue equivalents with enhanced physiological relevance and greater predictive accuracy. In summary, we present a highly characterised in vitro nasal mucosal construct that accurately reflects native microanatomy. Such technology will be of value to a wide range of applications, most notably, undertaking basic research and in vitro pharmaceutical screening, of which nasal mucosal models have become increasingly applicable due to the popularity of intranasal drug and vaccine delivery methods.

## Graphical abstract

Bioengineered tissue constructs are vital technologies employed in in vitro testing of both fundamental biological principles and industrial screening practices. Current approaches to modelling the nasal mucosa in vitro are not representative and are associated with limitations that impact their reliability and predictive value. In this study, we introduce a novel bioengineered human nasal airway construct that combines advanced 3D cell‐culture technology with a heterogeneous mix of tissue‐specific cell types that neosynthesise endogenous extracellular matrix and self‑organise to produce a sophisticated construct consisting of both stromal and epithelial compartments reminiscent of native human nasal mucosal tissue.

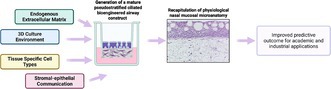

## 1 INTRODUCTION

The nasal epithelium constitutes one of the key elements of the upper respiratory tract termed the conducting zone. This area is functionally complex; in that, it provides a permeable barrier between the internal and external environments, delivering both physical and immunological protection (Druce, 1986; Harkema, 1991; Stucki et al., 2024). It is also responsible for absorbing water‐soluble and reactive gases, trapping inhaled particles, and is capable of metabolism of airborne xenobiotics (Brain, 1970; Lazaridis, 2023). As the biological concept suggests, cell and tissue structure is inherently linked to functionality; therefore, the microanatomy of the nasal epithelium is as equally diverse and complex as its range of functions. This poses challenges when developing in vitro model systems that aim to replicate in vivo functionality and emphasises the need for physiologically representative screening platforms capable of nasal drug and vaccine testing, particularly for respiratory infections.

The microanatomy of the nasal epithelium varies depending on location and function. The nasal cavity can be broadly divided into three regions, one being the vestibule, an internal continuation of the cutaneous lining of the external nose. The vestibule transitions into the respiratory region which is highly vascularised and therefore an attractive target for drug delivery, exhibiting effective permeability of molecules. The respiratory region is characterised at a cellular level through a heterogeneous population of varied cell types arranged as a pseudostratified epithelium, including columnar ciliated cells, columnar non‐ciliated cells, goblet cells and basal cells (Harkema et al., 2018; Reid et al., 2005). This complex cell population lends itself to a myriad of functions including clearance of particles, support, mucus secretion and tissue homeostasis and maintenance. The final region is the olfactory region, which is a specialised sensory tissue responsible for the modality of smell. This is reflected in its cellular composition with the presence of secretory goblet cells in the respiratory region replaced by olfactory receptor neurons capable of sensation (Pires et al., 2009).

The nasal mucosa, specifically the respiratory region, possesses a range of properties that make it an attractive drug delivery site, including a large surface area for absorption due to microvilli and a thin and highly vascularised structure with high blood flow rate. The direct transport of absorbed substances into systemic circulation is an attractive attribute as it circumvents some of the limitations associated with oral drug delivery. These properties also lend themselves to vaccine delivery, particularly targeted against respiratory infections such as influenza and, in recent years, SARS‐CoV‐2 (Altay Benetti et al., 2024; Davitt & Lavelle, 2015; Miquel‐Clopés et al., 2019; Sonvico et al., 2023; Yuki & Kiyono, 2009). Studies show that antigen delivery at the point of entry, when considering intranasal delivery for respiratory pathogens, results in enhanced protective immunity compared with intramuscular injection while eliciting long‐term systemic immunity (Brandtzaeg, 2010; Chavda et al., 2021; Fatuzzo et al., 2023; Gallichan & Rosenthal, 1996; Holmgren & Czerkinsky, 2005).

While the nasal delivery route has long since been used predominantly in the prevention, treatment or symptomatic relief of topical conditions such as congestion, allergies or infections, it is growing in popularity as a mechanism for systemic drug and vaccine administration due to its minimally invasive delivery mechanism (Ghori et al., 2015; Hussein et al., 2020; Illum, 2003; Keller et al., 2022; Kim et al., 2018; Ugwoke et al., 2001).

Given the immunological advantages, pain‐free administration, suitability for self‐medication and enhanced patient compliance, this route of drug delivery proposes a therapeutically viable alternative systemic drug delivery mechanism (De Martini et al., 2023; Ghori et al., 2015; Grassin‐Delyle et al., 2012; Hussein et al., 2020; Keller et al., 2022; Kim et al., 2018; Ugwoke et al., 2001). In the last decade, there has been an almost threefold increase in the number of clinical trials involving intranasal delivery systems, which is higher than that of other administration routes (Keller et al., 2022). Therefore, there is parallel demand for an in vitro model system that accurately recapitulates the structure and function of the nasal mucosa, capable of acting as a preclinical assessment tool.

Technological advancements within the field of bioengineering have seen an increase in the development of in vitro constructed tissues capable of modelling aspects of native tissue structure and function. These in vitro systems offer a more advanced alternative to traditional two‐dimensional (2D) cell culture techniques, and they are able to provide better predictive outcomes and increased precision, thereby streamlining the clinical trial process (Allcock et al., 2023; Devarasetty et al., 2018; Nam et al., 2015). Although bioengineered models of the nasal mucosa have been reported in the literature, they themselves are limited and often do not reflect the complex microanatomy and cellular interactions critical for tissue functionality in vivo.

Many nasal mucosal constructs described in both the literature and those that are commercially available only model the epithelial compartment of the tissue atop a stiff porous scaffold (Charles et al., 2019; Fonseca et al., 2025; Ladel et al., 2018; Luengen et al., 2020; Roberts et al., 2018). This approach fails to incorporate the complex interactions and communication between the stromal and epithelial compartments, which is a crucial factor responsible for normal tissue differentiation, physiological tissue homeostasis and functionality, and is often associated with suboptimal epithelial morphology (Folkerts & Nijkamp, 1998; Freer et al., 2024; Paulsson, 1992; Tam et al., 2011; Timpl, 1989; Wadsworth et al., 2004). Some full thickness nasal mucosal constructs have been reported; however, many utilise animal‐derived components which are inherently variable and introduce cross species compatibility considerations (Ndongo Sonfack et al., 2024; Wengst & Reichl, 2010), often rendering them inappropriate for human relevant studies. Most notably, the use of animal‐derived matrix components such as collagen I within the stromal compartment is a popular strategy; however, inherent batch‐to‐batch variability and limited extracellular matrix (ECM) composition impact biochemical interactions within such systems and reduce physiological relevance (Deniz Derman et al., 2023).

Other bioengineering approaches aimed to improve throughput and therefore industrial adoption, include organoid and tissue‐on‐a chip models. Organoid culture often involves the co‐culture of stromal and epithelial cells as a spherical structure in suspension culture. However, this can create a diffusion gradient resulting in the cells at the centre of the structure being starved of oxygen and nutrients leading to necrosis (Bonillo‐Lopez et al., 2025; Boyd et al., 2025; Chiu et al., 2022; Juarez‐Moreno et al., 2022; Li et al., 2024). Additionally, the spherical geometry of such cultures fails to recapitulate native tissue architecture, an essential fundamental principle known to impact functionality. The tissue‐on‐a‐chip approach not only lends itself to scaling up and increased throughput but also often incorporates the inclusion of additional, more complex microenvironmental factors such as airflow or fluid flow (Bendas et al., 2023; Koch et al., 2023; Walls et al., 2024). However, the actual tissue constructs cultured within these systems often recapitulates only the epithelial compartment cultured at the air–liquid interface engineered on a simple membrane, which as previously discussed, lacks the complex stromal–epithelial interactions that are essential for adequate tissue development, homeostasis and functionality (Brooks et al., 2022; Gholizadeh et al., 2023; Nof et al., 2022; Usman Khan et al., 2023).

Accordingly, there is a need for a physiologically representative human nasal mucosa model capable of providing useful preclinical, predictive data and to serve as a research tool for understanding fundamental cell and tissue mechanisms. In this study, we describe technology that enables tissue‐specific airway fibroblasts to neosynthesise endogenous ECM components. As demonstrated in other bioengineered tissue constructs (De Los Santos Gomez et al., 2024; Freer et al., 2023, 2024; Goncalves et al., 2022; Roger et al., 2019), this not only provides structural support to the overlying epithelial layer but also secreted factors from the fibroblasts enhance and support tissue development (Costello et al., 2024; Freer et al., 2024; Goncalves et al., 2022). We describe the generation of a fully humanised, robust, nasal mucosal construct consisting of a complex population of cell types that accurately reflect the anatomy native to the respiratory region of the nasal epithelium. We demonstrate the ability of this approach to recapitulate the aspects of the in vivo tissue microanatomy, characterised at an ultrastructural level, with the presence of junctional complexes, ECM, cilia, secretions and cellular interactions representative of the native physiological environment. Moreover, this introduces a well‐characterised platform technology capable of significant insights within the pharmaceutical field.

## 2 MATERIALS AND METHODS

### 2.1 Maintenance of human cell lines

Primary human nasal airway epithelial cells isolated from healthy human nasal biopsies (hAECN, Epithelix, Geneva, Switzerland) were maintained in PneumaCult™‐Ex Plus Basal Medium supplemented with 10 mL PneumaCult™‐Ex Plus 50X Supplement and 0.5 mL Hydrocortisone Stock Solution as per the manufacturer's instructions (STEMCELL Technologies, Cambridge, UK). hAECN were maintained at 37°C and 5% CO_2_ in a humidified environment until they reached 80% confluency, at which point they were used in experiments.

Human foetal lung‐derived fibroblasts, MRC‐5 pd25 (European Collection of Authenticated Cell Cultures, ECACC, Salisbury, UK) were maintained in Minimum Essential Medium (MEM, ThermoFisher Scientific, Loughborough, UK) with non‐essential amino acids supplemented with 10% Foetal Bovine Serum (FBS, ThermoFisher Scientific) and 2 mM L‐glutamine (ThermoFisher Scientific). Cells were maintained at 37°C and 5% CO_2_ in a humidified environment at a density of 2.8 × 10^3^ cm^−2^ until they reached 80% confluency, at which point they were used in experiments.

### 2.2 Epithelial‐only (EO) tissue construction

Epithelial‐only nasal constructs were generated by seeding hAECN onto polyester Transwell® (Sigma‐Aldrich, Cambridge, UK) cell culture inserts (6.5 mm diameter, 0.4 μm pore size) and cultured in standard 24‐well culture plates. Cells were seeded at a density of 0.25 × 10^6^ cells per insert with a volume of 200 μL supplemented PneumaCult™‐EX Plus medium per insert, 500 μL of medium was added to the basal compartment surrounding the insert and media were changed after 24 h post‐seeding. Epithelial‐only models were then raised to the air–liquid interface (ALI) 4 days post‐seeding. Medium was aspirated from the model apical surface and 500 μL of PmeumaCult™‐ALI Basal Medium containing PneumaCult™‐ALI 10× supplement (STEMCELL Technologies) with PneumaCult™‐ALI maintenance supplement, heparin solution and hydrocortisone added as per manufacturer's instructions (STEMCELL Technologies) was added to the basal compartment. Epithelial‐only models were then maintained at 37°C and 5% CO_2_ in a humidified environment for 21 days to form a mature tissue construct with medium changes every 2–3 days.

### 2.3 Submucosal compartment construction

Submucosal compartments were generated by seeding MRC‐5 pd25 fibroblasts into Alvetex® Scaffold (REPROCELL Europe Ltd., Glasgow, UK), a porous, polystyrene, inert scaffold used routinely for bioengineering applications. Twenty‐four‐well format Alvetex® Scaffold inserts were rendered hydrophilic through a brief incubation in 70% (v/v) ethanol for 5 min, prior to washing in phosphate‐buffered saline (PBS) twice. Fibroblasts were added to each insert at a density of 0.5 × 10^6^ cells per insert in 100 μL MEM + non‐essential amino acids, supplemented with 10% FBS and 2 mM L‐glutamine. This seeding density is consistent with our skin and intestinal bioengineered constructs and when maintained for up to 28 days and is known to support an overlying epithelial compartment without cellular invasion into the submucosal compartment (Costello et al., 2021; Darling et al., 2020; Freer et al., 2024; Roger et al., 2019). Cells were incubated for 2 h at 37°C and 5% CO_2_ in a humidified environment to promote attachment before 4 mL medium was added to each insert, supplemented with 100 μg mL^−1^ ascorbic acid (Sigma‐Aldrich) and 5 ng mL^−1^ TGFβ1 (ThermoFisher Scientific) to promote ECM neosynthesis. Cultures were maintained for 28 days and media were replenished twice weekly.

### 2.4 Full thickness (FT) nasal tissue construction

Submucosal compartments were generated as described in the previous section. hAECN were then seeded onto 28‐day mature submucosal compartments at a density of 3 × 10^5^ cells per insert, in a volume of 100 μL PneumaCult™‐Ex Plus Medium. Cells were incubated for 2 h at 37°C and 5% CO_2_ in a humidified environment to promote attachment, and 4 mL PneumaCult™‐Ex Plus Medium was then added to each insert. Following initial cell seeding, medium was replenished after 24 h. After a 3‐day incubation period in submerged culture conditions, tissue constructs were raised to the ALI. Medium was aspirated from the apical surface and 2 mL PneumaCult™‐ALI Medium (supplemented as described in EO tissue construction section) was added to the basal compartment. Tissue constructs were maintained in culture and allowed to mature for up to 28 days, and media were replenished twice weekly.

### 2.5 Human tissue

The human nasal tissue sample used in this study was ethically sourced via the REPROCELL Tissue Network (approved as a research tissue bank by the West of Scotland Research Ethics Committee, 17/WS/0049). All donors or approved persons on their behalf provided written informed consent for tissue donation and its use in biological or medical research. Tissue was transferred to Durham University (Licensing Number: 12382) under a Material Transfer Agreement (MTA).

### 2.6 Paraffin wax embedding

Samples were embedded in paraffin wax as previously described (Costello et al., 2023, 2024; Goncalves et al., 2022). Both engineered tissue constructs and human tissue were fixed in 10% formalin (Sigma‐Aldrich). Following this, samples were gradually dehydrated in ethanol (30%–100%) and incubated in Histoclear (Scientific Laboratory Supplies, Nottingham, UK) followed by a 1:1 mix of Histoclear and molten paraffin wax (ThermoFisher Scientific). Samples were then finally incubated in molten paraffin wax alone, before being embedded in plastic moulds (CellPath, Newton, UK). Paraffin wax blocks were sectioned transversely at 5 μm using a microtome (Leica RM2125RT) and mounted onto charged microscope slides (ThermoFisher Scientific).

### 2.7 Haematoxylin & eosin (H&E) staining

Samples were deparaffinised in Histoclear and rehydrated through a graded ethanol series (100%–70%). Samples were then incubated in Mayer's haematoxylin (Sigma‐Aldrich) for 5 min followed by alkaline ethanol to blue the nuclei for 30 s. Samples were then dehydrated through a series of progressive ethanol baths (70%–95%) and counterstained in eosin (Sigma‐Aldrich) for 30 s before final dehydration. Slides were then cleared in Histoclear and mounted with Omnimount (Scientific Laboratory Supplies).

### 2.8 Periodic acid–Schiff (PAS) staining

A periodic acid–Schiff with Alcian Blue staining kit (Atom Scientific, Greater Manchester, UK) was used as per the manufacturer's instructions to detect mucosubstances. Samples were deparaffinised in Histoclear and rehydrated through a graded ethanol series (100%–70%) prior to staining with Alcian Blue for 10 min. Slides were then washed in deionised water (dH_2_O) and treated with 1% periodic acid solution for 10 min before washing again in dH_2_O. Slides were then incubated in Schiff reagent for 10 min, washed in dH_2_0 and nuclei were counterstained in Haemalum Mayer for 30 s. Finally, slides were washed and differentiated in 0.5% acid alcohol and blued in tap water prior to a final dehydration gradient of ethanols, cleared in Histoclear and mounted with Omnimount.

### 2.9 Immunofluorescence

Samples were deparaffinised in Histoclear and rehydrated through a graded ethanol series (100%–70%). Antigen retrieval was performed in citrate buffer pH 6 (Sigma‐Aldrich) at 95°C for 20 min. Samples were then blocked and permeabilised for 60 min at room temperature in a solution of 20% neonatal calf serum (NCS, Sigma‐Aldrich) and 0.4% Triton X‐100 (Sigma‐Aldrich) diluted in PBS. Samples were then incubated with a primary antibody (Table S1) diluted in blocking buffer overnight at 4°C. Slides were then washed three times in PBS and incubated with the appropriate fluorescently labelled secondary antibody (Table S1) for 60 min at room temperature. Following incubation, slides were washed three times in PBS containing Hoechst 33342 diluted at 1:10,000 (ThermoFisher Scientific) and mounted using Vectashield (Vector Laboratories, Peterborough, UK).

### 2.10 Light microscopy

Histological stains including H&E, PAS and Toluidine Blue staining were imaged using a Leica ICC50 high‐definition camera and bright‐field microscope, and images were captured using the Leica EZ software.

Immunofluorescence images were captured using the Zeiss 880 confocal microscope with Airyscan Zen software.

### 2.11 Transmission electron microscopy (TEM)

Samples were prepared for TEM as previously described (Roger et al., 2019). This involved fixation in Karnovsky's fixative (8% paraformaldehyde, 25% glutaraldehyde, 0.2 M cacodylate buffer (Agar Scientific, Rotherham, UK) and dH_2_O). Samples were then washed in 0.1 M cacodylate buffer (pH 7.6) and further fixed for 60 min in a solution of 2% osmium tetroxide (Agar Scientific) and 0.2 M cacodylate buffer. Samples were then washed once again in 0.1 M cacodylate buffer and dehydrated through a series of ethanol baths before being embedded in Agar Epon Resin (Agar Scientific). This involved incubations in a series of solutions consisting of 100% ethanol and propylene oxide (1:1), propylene oxide alone, propylene oxide and Agar 100 Epon Resin (1:1), and finally Agar 100 Epon Resin alone. Samples were then embedded in Epon Resin and allowed to polymerise at 60°C for 24 h.

Once embedded, ultrathin sections were cut at 70 nm using a diamond knife (Agar Scientific) on a Reichert Ultracut S Ultramicrotome (Leica) and transferred to 200 mesh copper formvar‐coated grids (Agar Scientific). Samples were then stained with 1% uranyl acetate (VWR International, Leicestershire, UK) in 70% ethanol and lead citrate (VWR International) prior to visualisation and image capture on a Hitachi H7600 Transmission Electron Microscope.

### 2.12 Toluidine blue staining

Ultrathin sections were captured onto microscope slides and stained in 1% toluidine blue for 1 min. Samples were then washed in dH_2_O before being mounted using Distyrene, Plasticizer and Xylene (DPX) (Sigma‐Aldrich) and imaged.

### 2.13 Scanning electron microscopy (SEM)

Samples were prepared for SEM as previously described (Roger et al., 2019). Samples were critically dried (BAL‐TEC CPD 030, Leica) and coated with 5 nm platinum in a Cressington Coating System 328 (Cressington, Merseyside, UK). Sample visualisation was performed using a S5200 scanning electron microscope (Hitachi, Birmingham, UK).

### 2.14 Reproducibility

In order to evidence the robust nature of the tissue construct described in this study, we conducted each experiment in triplicate with a minimum of three constructs engineered from a population of cells within a given experiment. Each experiment was then repeated independently three times. Therefore, a total of nine constructs were analysed per technique described. This ensured the reproducibility of the engineered tissue constructs and allowed for confident conclusions to be drawn.

## 3 RESULTS

### 3.1 Optimisation of stromal and epithelial compartments in isolation

**FIGURE 1:**
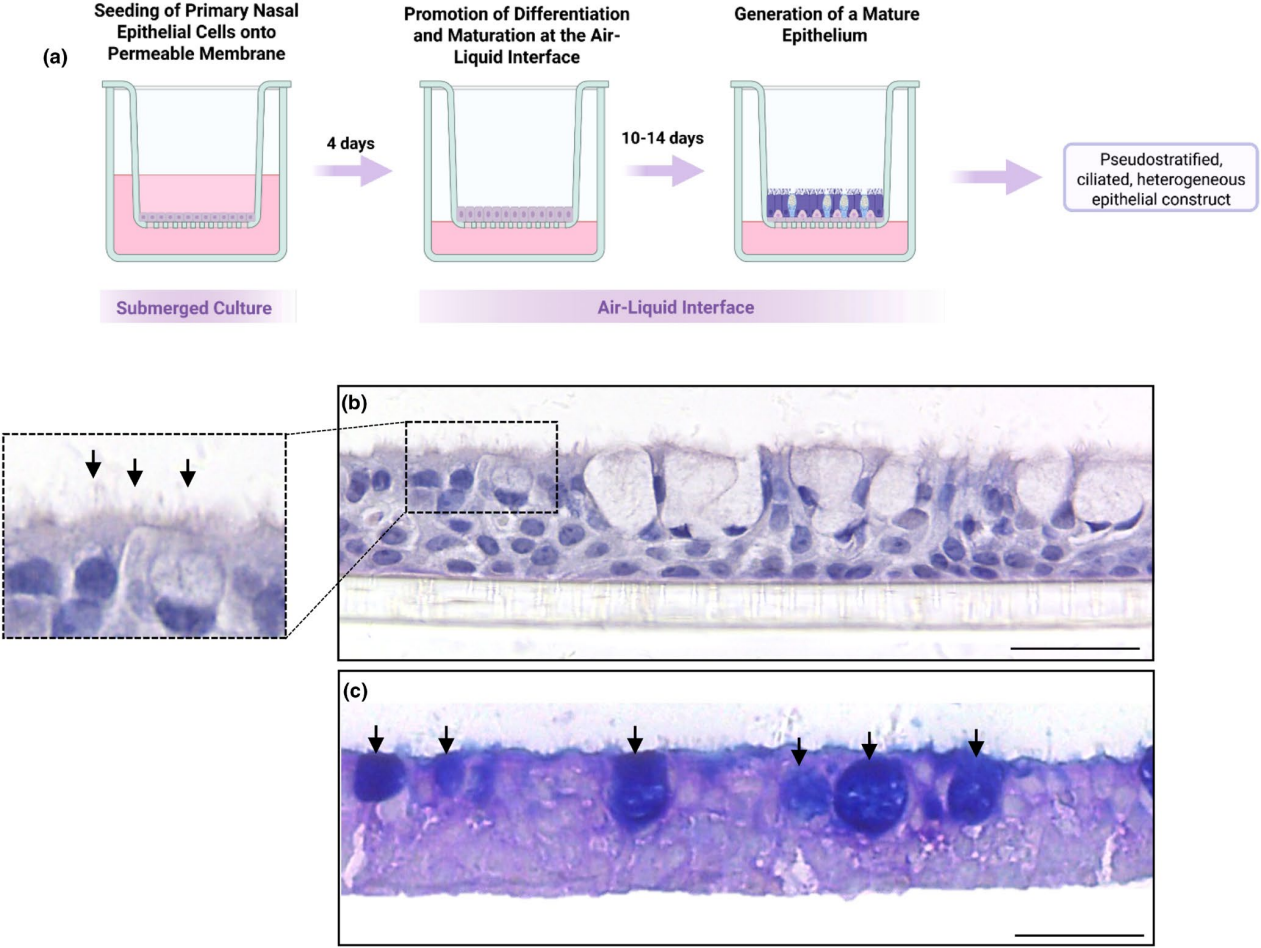
Bioengineering of a ciliated epithelial compartment in isolation. Schematic representation (a) of the culture methodology employed to create a heterogeneous epithelium‐only construct from primary nasal airway epithelial cells (hAECN), which were seeded onto a permeable membrane for 4 days in submerged culture prior to raising to the air–liquid interface for a further 10–14 days to promote cellular differentiation. H&E staining (b) reveals resultant constructs appear pseudostratified and heterogeneous in nature. Cilia are identifiable on the apical surface of the construct (arrows). Periodic acid–Schiff (c, PAS) staining demonstrates the presence of mucins stained blue within goblet cells (arrows). Scale bars: b, c = 50 μm.

The nasal epithelium in vivo comprises a heterogeneous cell population that forms a pseudostratified, ciliated interface between the internal and external environments. To engineer the epithelial compartment of this tissue in vitro, a commercially available primary cell population (hAECN) was seeded onto a polyester membrane for 4 days in submerged culture. With the purpose of promoting differentiation of the cells, they were then raised to the air–liquid interface for a further 21 days, which resulted in the generation of a pseudostratified epithelial tissue construct (Figure 1a). Histological analysis revealed the structure of the EO construct (Figure 1b), to be that of a pseudostratified heterogeneous epithelium, with a clearly ciliated surface (arrows). Furthermore, PAS staining (Figure 1c) demonstrated the presence of mucins (stained blue) within globules present in the tissue. This is indicative of goblet cell activity and thereby acts as evidence to suggest that the construct consists of multiple functional cell types.

**FIGURE 2:**
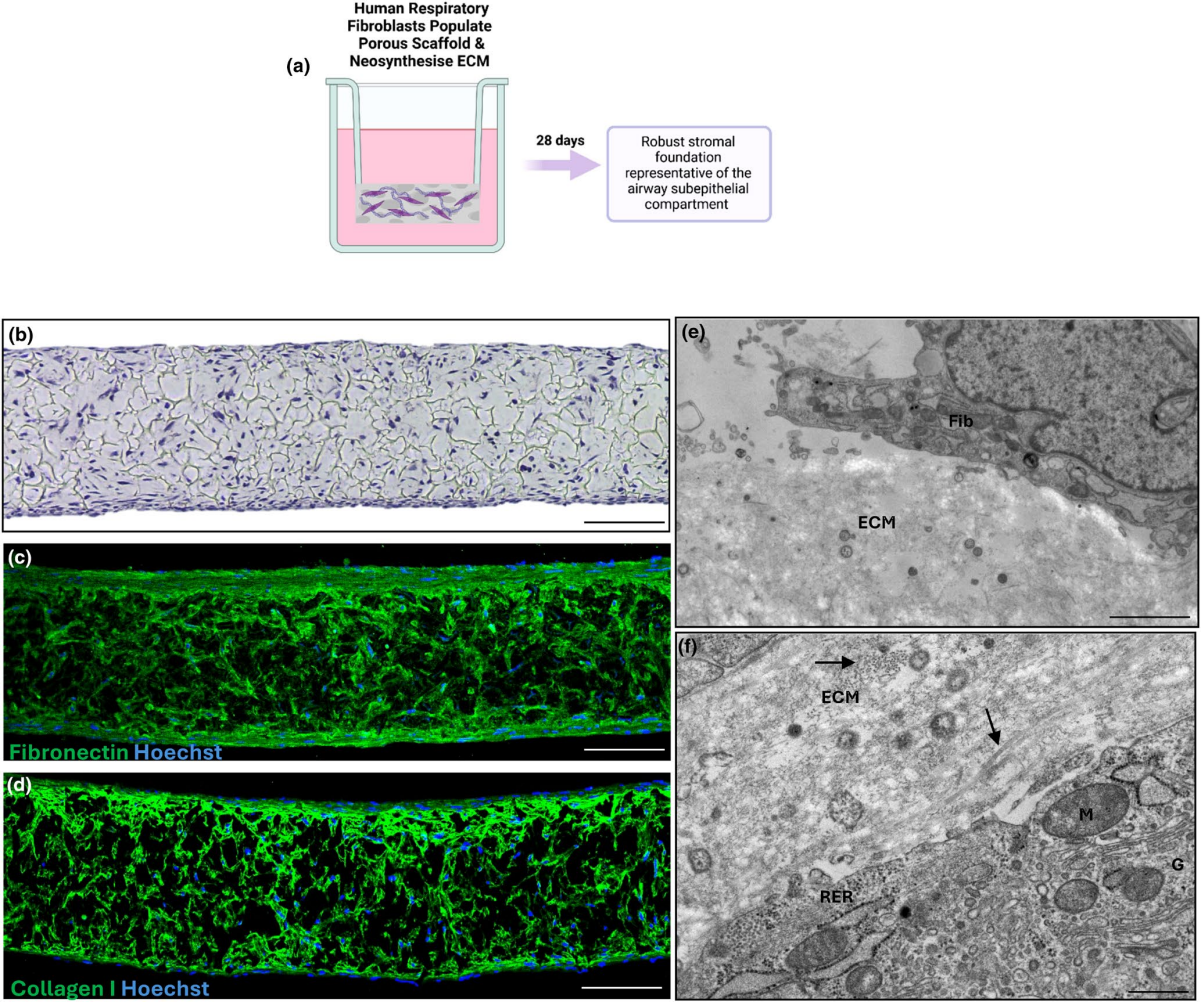
Bioengineering an extracellular matrix‐rich stromal compartment in isolation. Schematic representation (a) depicting the culture of MRC‐5 pd25 fibroblasts within a porous polystyrene scaffold for 28 days to engineer a robust submucosal compartment, rich in endogenous extracellular matrix (ECM). Representative H&E staining (b) demonstrates the population of the scaffold by the fibroblasts, while immunofluorescence images demonstrate significant deposition of fibronectin (green, c) and collagen I (d) (green, nuclei stained blue by Hoechst). Representative transmission electron micrographs reveal significant ECM deposition, surrounding a resident fibroblast (e), with both longitudinal and transverse bundles of collagen fibres visible (f, arrowheads). Scale bars: b–d = 100 μm, e = 2 μm, f = 500 nm. ECM, extracellular matrix; Fib, fibroblast; G, golgi apparatus; M, mitochondria; RER, rough endoplasmic reticulum.

The generation of a robust stromal foundation is an essential step in the construction of any bioengineered epithelial tissue. For this reason, we initially optimised the growth of human respiratory fibroblasts within the three‐dimensional (3D) culture environment throughout a 28‐day period to promote tissue maturity and deposition of endogenous ECM (Figure 2a). Histological analysis (Figure 2b) revealed that the scaffold contained viable fibroblasts, which can be seen throughout the entire depth of the 3D material, while also lining the apical and basal surfaces. The density of the fibroblast population throughout the 3D material compared favourably to other bioengineered tissue constructs (Costello et al., 2019; Darling et al., 2020; Freer et al., 2024; Roger et al., 2019), which were able to support the construction of an epithelial compartment without invasion into the submucosal compartment.

Deposition of endogenous, tissue‐specific, human ECM by fibroblasts has proven crucial to the successful development of bioengineered intestine and skin full thickness platforms (Freer et al., 2024; Goncalves et al., 2022; Roger et al., 2019). This eliminates the need for exogenous, animal‐derived components and increases the physiological relevance of the tissue, while promoting communication between the tissue compartments. Immunofluorescence reactivity demonstrates that the human respiratory fibroblasts cultured within this system were able to produce and deposit both fibronectin (Figure 2c) and collagen I (Figure 2d) in significant quantities within the scaffold; expression of fibronectin and collagen I is consistent with ECM derivatives expressed within the nasal epithelium in vivo (Bao et al., 2021). At an ultrastructural level, the organisation of extracellular fibres consistent with the ECM is clearly visible (Figure 2e). ECM proteins neosynthesised by the fibroblasts have formed a dense network of longitudinal and transverse fibres that provide physical and biochemical support to the cellular population (Figure 2f).

### 3.2 Development of a full thickness nasal mucosal construct that recapitulates aspects of native tissue structure

**FIGURE 3:**
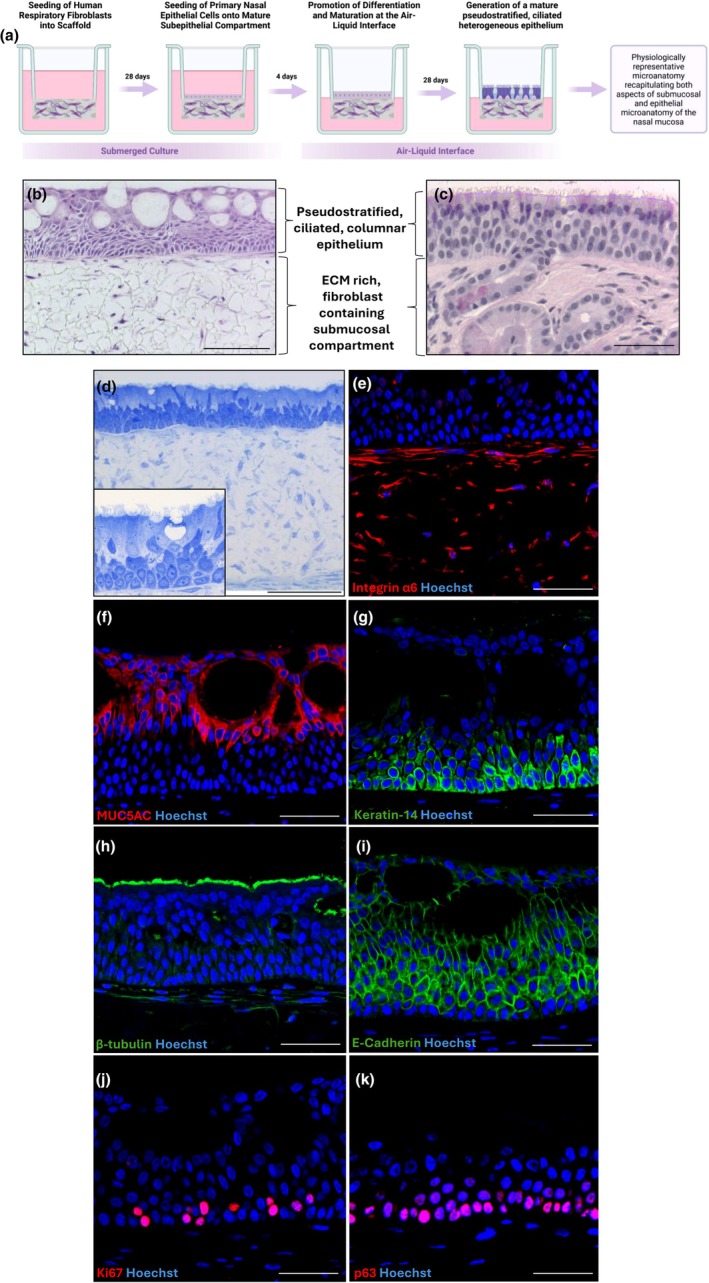
A bioengineered full thickness nasal epithelium construct that recapitulates native tissue architecture. Schematic representation of the culture process (a), whereby fibroblasts were seeded within the scaffold, which then proliferate and neosynthesise extracellular matrix (ECM) over 28 days to form a robust submucosal compartment. Primary nasal airway epithelial cells (hAECN) were then seeded onto the construct and allowed to proliferate in submerged culture for 4 days, before being raised to the air–liquid interface to promote differentiation and formation of the mature construct. Representative H&E staining reveals morphological similarities between the bioengineered nasal construct (b) and native human nasal respiratory tissue (c). Both appear as a pseudostratified tissue consisting of columnar, ciliated cells atop an ECM‐rich submucosal compartment. Toluidine blue staining (d) reveals a higher magnification visualisation of cilia present on the apical surface of the engineered epithelium. Immunofluorescence staining for biomarkers consistent with nasal epithelial tissue structure demonstrates expected expression and distribution: integrin α6 (e, red), MU5AC (f, red), keratin‐14 (g, green), β‐tubulin (h, green), E‐cadherin (i, green), Ki67 (j, red) and p63 (k, red), with nuclei highlighted by Hoechst in blue. Scale bars: b, d = 100 μm, c, e–k = 50 μm.

Following the development and characterisation of separate stromal and epithelial compartments, methods were optimised to create a single mucosal construct to develop a full thickness platform reminiscent of the native tissue (Figure 3a). Histological analysis demonstrated that the resulting tissue construct (Figure 3b) bore similarities to the native tissue (Figure 3c), exhibiting a pseudostratified, ciliated, columnar epithelial layer, atop an ECM‐rich, fibroblast‐containing stromal compartment. In addition, the epithelial compartment comprised a heterogeneous cell population, with the presence of mucin globules identifiable from the histological images. Toluidine blue staining of ultrathin sections of the full thickness bioengineered tissue (Figure 3d) provides a more in‐depth histological viewpoint, with cilia having visibly lined the apical surface of the epithelium. The morphology of individual cells showed the construct containing a combination of both columnar ciliated cells and cuboidal basal cells.

Overall, the structure of the bioengineered nasal epithelial tissue compared favourably to that of the native tissue. Interestingly, the epithelial thickness in the full thickness construct described in Figure 3 was substantially increased compared with the epithelial only construct described in Figure 1, a finding consistent across all of the constructs analysed within this study. Similarly, the number of distinct epithelial cells that exhibited a columnar morphology was also increased in the full thickness construct, emphasising the importance of stromal support in epithelial development and homeostasis.

Expression of tissue‐specific biomarkers was also assessed to characterise and demonstrate alignment of expression patterns with the anticipated expression within the native tissue. Expression of integrin α6 is ubiquitous among fibroblasts within the stromal compartment (Figure 3e), both throughout the scaffold and at the epithelial–stromal junction. This is consistent with the in vivo functionality of integrin α6, which is responsible for key interactions between fibroblasts and ECM components, including mechanosensing and developmental regulation; furthermore, integrin α6 is also known to interact with the basement membrane in epithelial tissues (Chen et al., 2016; Ma et al., 2023; Sonnenberg et al., 1991).

**FIGURE 4:**
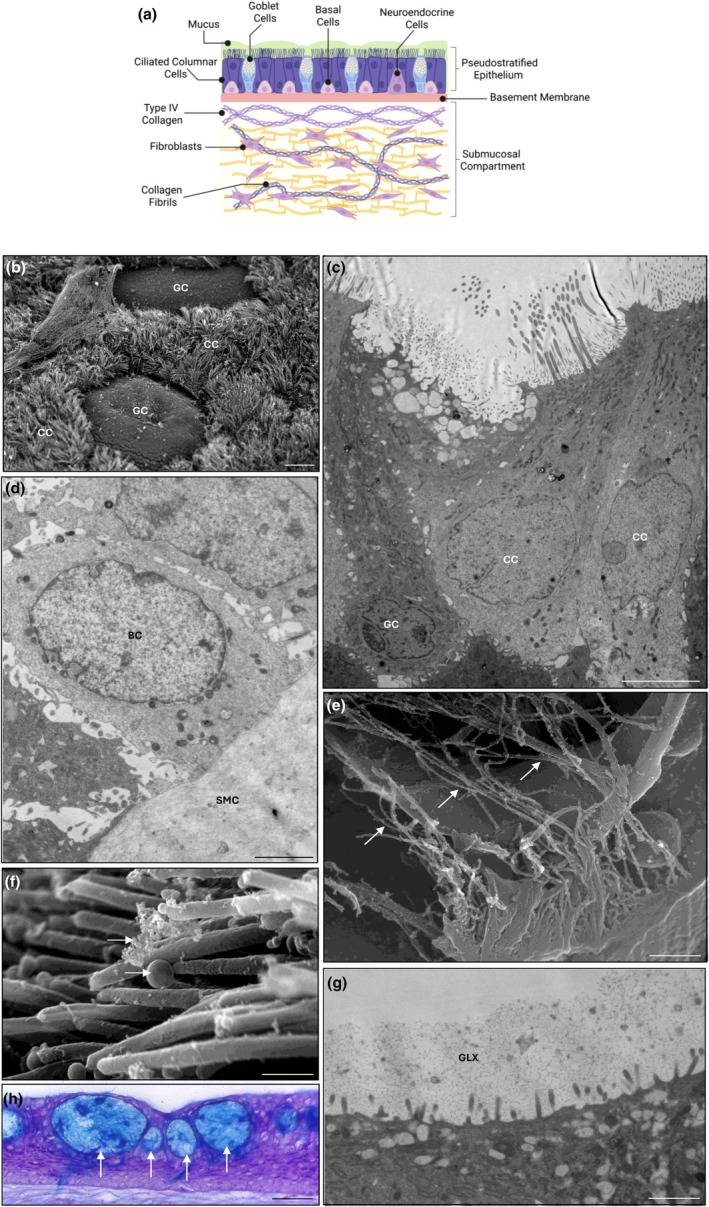
Structure of the bioengineered respiratory epithelium is consistent with the microanatomy of the native human nasal tissue. Schematic (a) illustrating the anatomy of nasal mucosal tissue: a heterogeneous cell population inclusive of ciliated columnar cells, basal cells, goblet cells and neuroendocrine cells which form a pseudostratified epithelium, separated from the submucosal compartment by the basement membrane. Representative scanning electron micrograph (b) shows the presence of both ciliated cells (CC) and goblet cells (GC) situated across the surface of the bioengineered tissue construct. Transmission electron micrographs of transverse tissue cross‐sections depict the presence of both ciliated and goblet cells adjacent to one another (c) along with basal cells (d, BC). Secretions consistent with localisation of the glycocalyx (GLX) within the tissue are identified both on the surface of the construct by scanning electron microscopy (f) where they can be located between cilia (arrows) and by transmission electron microscopy (g) which depicts a diffuse coating on the model surface. Periodic acid–Schiff (PAS) staining also demonstrates the presence of mucins within deposits secreted by goblet cells located within the epithelial layer of the construct (h, arrows). Scale bars: b = 10 μm, c, f = 5 μm, d = 2 μm, e = 200 nm, g = 1 μm, h = 25 μm. BC, basal cell; CC, ciliated cell; GC, goblet cell; GLX, glycocalyx; SMC, submucosal compartment.

Expression of MUC5AC was detectable in the suprabasal area of the epithelium (Figure 3f), particularly around large mucin globules as identified through histological analysis. This is consistent with expected expression as MUC5AC is a mucin localised within the respiratory tract, whose function is to trap and protect the lungs from inhaled contaminants and pathogens (Jaramillo et al., 2018; Song et al., 2025). It was interesting to note the accumulation of mucin deposits within the upper half of the epithelium, creating large, internalised vacuoles occupied by secreted material (Figures 3d and 4h).

Similarly, other biomarkers associated with epithelial structure were identified in the bioengineered nasal construct. These include keratin‐14 (Figure 3b,d), a cytoskeletal component expressed in dividing basal cells (Ievlev et al., 2023), β‐tubulin (Figure 3h), a cytoskeletal component of cilia (He et al., 2020), expressed on the apical surface of the tissue consistent with cilia identification from toluidine blue staining, and E‐cadherin (Figure 3i), a junctional component (Nawijn et al., 2011), expressed throughout the epithelial compartment.

In addition to structural components, expression of biomarkers associated with epithelial homeostasis was another important consideration in the characterisation of this bioengineered construct. As such, robust expression of the proliferation marker ki67 was identified in the basal region of the epithelium (Figure 3j), like expression of p63, a transcription factor responsible for the development of a stratified epithelium (Figure 3k).

Collectively, these data support the notion that the bioengineered nasal mucosal construct morphology accurately recapitulates aspects of native tissue anatomy. This is inclusive of expected biomarker expression of proteins consistent with epithelial function, tissue maintenance and structure. An ultrastructural level of characterisation that assesses the microanatomical structure of bioengineered constructs is, however, notably absent from alternative models reported in the literature (Capuana et al., 2022; Ladel et al., 2019; Ndongo Sonfack et al., 2024; Wengst & Reichl, 2010).

### 3.3 Ultrastructural recapitulation of nasal mucosal microanatomy in a bioengineered tissue construct

The mucosa found in the respiratory region of the nasal cavity comprises an epithelial compartment separated from a submucosal or stromal compartment by the basement membrane (Figure 4a). The epithelium consists of multiple cell types in a pseudostratified arrangement, including ciliated columnar cells, basal cells, neuroendocrine and goblet cells—which secrete mucus that lines the surface of the tissue. The stromal compartment consists of fibroblasts that secrete a dense network of ECM, along with structural proteins, and supporting cell types. To better assess the presence of these features associated with the epithelial compartment, we conducted analysis at an ultrastructural level using electron microscopy.

Scanning electron microscopy was utilised to visualise the surface of tissue constructs, revealing a heterogeneous topography comprised of cilia lining the surface of columnar cells and distinct goblet cells identifiable by their smooth apical surface and notable absence of cilia (Figure 4b). Cross sections of tissue constructs analysed via transmission electron microscopy confirm these findings (Figure 4c,d). Columnar cells with ciliated projections located on the apical surface reside adjacently to electron dense goblet cells and were identifiable through the presence of secretory vesicles and globules (Figure 4c). Basal cells possessing a more cuboidal morphology were also observed to line the basal region of the epithelial compartment, immediately adjacent to the submucosal compartment (Figure 4d).

The data show evidence of alternative cell types present in the epithelial compartment through differences in their morphology and corresponding functionality. For example, the staining of mucin and secretions that lined the surface of the tissue construct indicates activity of the goblet cells releasing their cargo. SEM micrographs depict secreted substances, the appearance of which is consistent with glycocalyx having formed a protective network across the surface of the construct (Figure 4e, arrows) and globules identifiable among cilia consistent with mucin secretion (Figure 4f, arrows). PAS staining also confirms the presence of mucins at a histological level through blue staining of globules within the tissue (Figure 4h, arrows).

Similarly, other cell surface specialisations in the form of microvilli were also identifiable, and branching molecules, likely to be carbohydrate of glycocalyx, were seen extending outward from the plasma membrane (Figure 4g). The production and secretion of glycocalyx is a particularly important aspect of respiratory physiology to capture in vitro, as not only does it provide protection and lubrication in vivo, but is also an important mediator of cellular signalling (Möckl, 2020; Ochs et al., 2020; Rizzo & Schmidt, 2023; Timm et al., 2023), therefore, its structural presence is also suggestive of physiologically relevant function within the epithelium.

**FIGURE 5:**
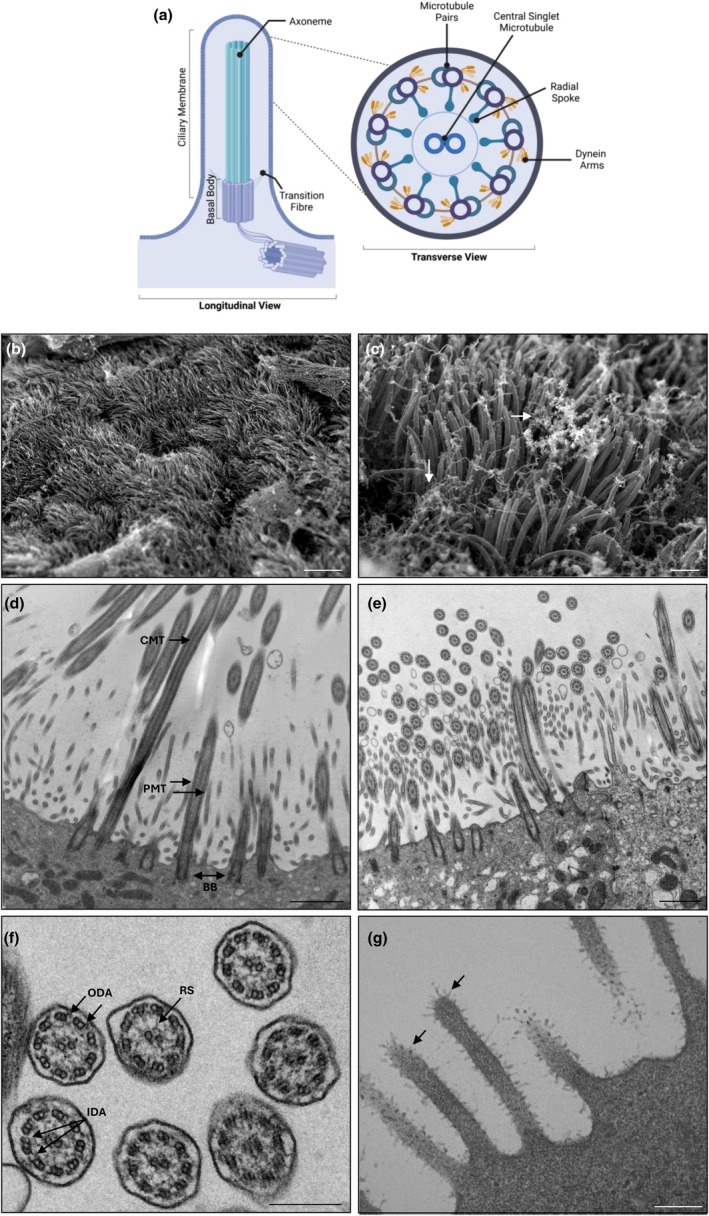
Ultrastructural analysis allows for identification of motile cilia and microvilli in bioengineered full thickness nasal epithelial construct. Schematic (a) illustrates the subcellular structure of motile cilia present within the respiratory tract. The axoneme can be identified longitudinally, while a transverse view demonstrates outer microtubule pairs, surrounding a central singlet microtubule. Representative scanning electron micrographs depict the presence of cilia on the surface of the epithelial tissue construct both at low (b) and high magnification (c), with mucus identifiable within the cilia (arrowheads). Transmission electron micrographs show the ultrastructural microanatomy within individual cilia (d) with central microtubules (CMT), peripheral microtubules (PMT) and basal bodies (BB) all visible. Both longitudinal and transverse sections of cilia can be identified within tissue cross sections (e). Ultrastructural analysis reveals typical motile cilia structure in the bioengineered construct, with the expected ‘9 + 2’ arrangement of microtubules (f), outer dynein arms (ODA), inner dynein arms (IDA) and radial spokes (RS) all identifiable. Similarly, the apical surface of goblet cells is decorated with microvilli coated with glycocalyx molecules (g) extending outwards from the plasma membrane (arrows) in transmission electron micrographs. Scale bars: b = 10 μm, c–e = 1 μm, f = 250 nm, g = 200 nm. BB, basal body; CMT, central microtubule; IDA, inner dynein arm; ODA, outer dynein arm; PMT, peripheral microtubule; RS, radial spoke.

A prominent feature of the respiratory epithelium is the presence of motile cilia whose functions include mucus transport, protection and fluid transport (Kuek & Lee, 2020; Satir & Christensen, 2007; Stannard & O'Callaghan, 2006). The ultrastructure of motile cilia is distinct from that of primary cilia which are immobile. Motile cilia typically exhibit a ‘9 + 2’ axonemal structure with two central single microtubules surrounded by nine outer microtubule doublets with additional accessory structures (Figure 5a). The axoneme features a basal body that anchors the cilia within the cytoplasm but also plays a pivotal role in cilia assembly (Hoyer‐Fender, 2013).

The presence of cilia lining the apical surface of the bioengineered construct is consistent with the expected topography of the respiratory nasal tract in vivo. This is demonstrated through the identification of cilia present on the apical surface of the construct via SEM at both low (Figure 5b) and high magnification (Figure 5c), with the presence of glycocalyx also discernible between cilia. The internal structure of cilia was analysed by TEM at an ultrastructural level and revealed the expected microanatomy consistent with motile cilia. Longitudinal cross sections exposed the presence of central microtubules located medially to peripheral microtubules, with basal bodies identifiable at the cilia base (Figure 5d).

Both longitudinal and transverse cross sections of cilia are visible in low magnification images, demonstrating the high density of cilia present on the surface of the tissue construct (Figure 5e). Transverse cross sections exhibit the typical ‘9 + 2’ arrangement of microtubules surrounding a central pair of single microtubules, consistent with motile cilia (Figure 5f). Ultrastructural details can be observed at this level including radial spokes connecting the central microtubule pair to outer doublets and hook‐shaped inner and outer dynein arms responsible for the generation of ciliary motion. These hallmarks are consistent with the ultrastructural features of functional, motile kinocilia (Pinto et al., 2020).

Similarly, tissue cross sections not only reveal the presence of cilia but also allow for the identification of microvilli on the surface of columnar cells (Figure 5g) with glycocalyx protrusions from the plasma membrane (arrows). Together, these surface specialisations not only highlight recreation of native tissue anatomy but also provide evidence to establish a level of functionality consistent with cellular structures in vivo.

**FIGURE 6:**
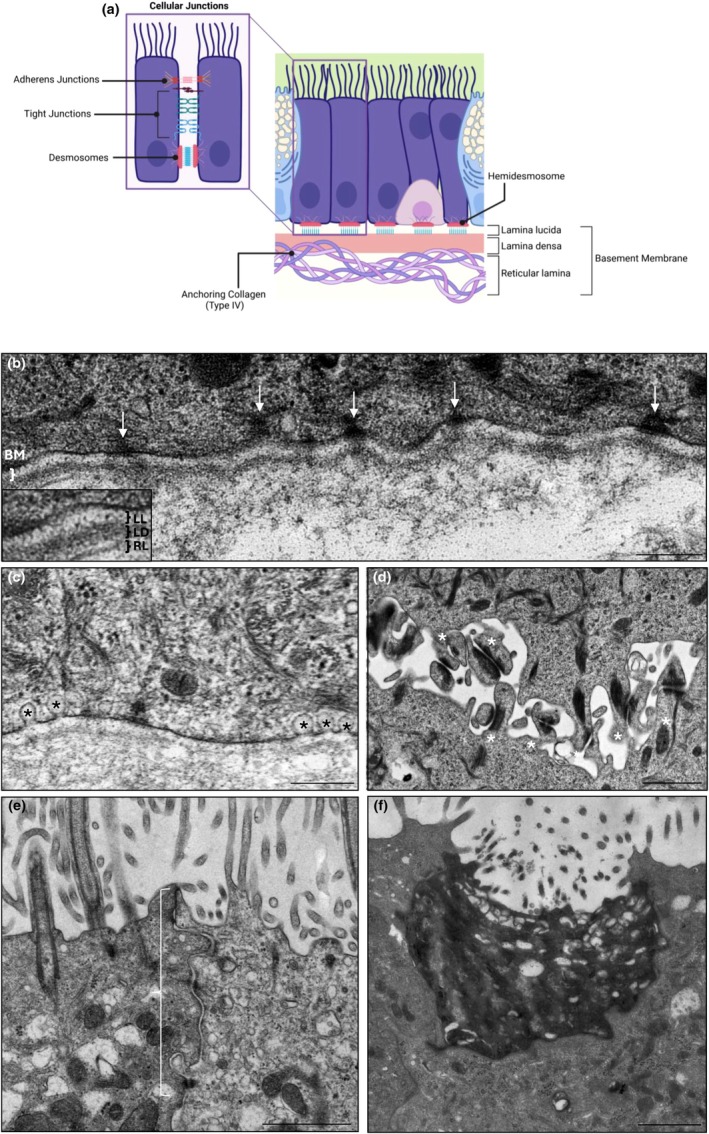
Formation of a trilayered basement membrane separating epithelial and submucosal compartments within the bioengineered nasal construct. Schematic representation (a) of intercellular junctional complexes, along with the trilayer structure of the basement membrane consisting of the lamina lucida, lamina densa and reticular lamina along with hemidesmosomes that anchor epithelial cells to the submucosal compartment. Ultrastructural analysis (b) reveals the presence of a trilayered basement membrane (BM) that separates epithelial and submucosal compartments within the bioengineered tissue, with hemidesmosomes (arrows) identifiable along its length, anchoring epithelial cells to the basement membrane. The individual components of the trilaminar structure of the basement membrane were also identifiable in transmission electron micrographs: lamina lucida (LL), lamina densa (LD), reticular lamina (RL). Representative transmission electron micrograph (c) demonstrates budding caveolae (*) mediating communication between the two tissue compartments, supporting the notion that not only the microanatomy is recapitulated in this tissue construct but also the signalling events that are essential for tissue homeostasis. Desmosomes (d, *) are identifiable between adjacent cells, with apical junctional complexes evident between neighbouring ciliated cells (e, arrow heads), and between ciliated and goblet cells (f, arrow heads). Scale bars: b, c = 250 nm, d–f = 1 μm. BM, basement membrane; LD, lamina densa; LL, lamina lucida; RL reticular lamina.

Communication between cells and tissue compartments is essential in the development and maintenance of any tissue; this is particularly the case in epithelial tissues, with support from the underlying stromal compartment playing an integral role in tissue homeostasis. The two compartments are separated by the basement membrane, which itself is comprised of three layers: lamina lucida, lamina dense and the reticular lamina, with cells anchored by hemidesmosomes located on the basal surface. Evidence shows that cells are anchored to one another via cellular junctions such as adherens and tight junctions alongside desmosomes, with such structures governing permeability and barrier function of the epithelium (Figure 6a).

The tri‐laminar structure of the basement membrane is clearly observed within the tissue construct via high magnification TEM (Figure 6b). Given that no exogenous ECM components were used in the construction of this bioengineered tissue, the cell types have self‐organised and the basement membrane has formed de novo through dynamic interactions between the epithelial cells and underlying fibroblasts located in the stromal compartment. The basement membrane comprises the electron dense plasma membrane, the electron lucent lamina lucida, the electron dense lamina densa and the more diffuse reticular laminar, all of which are consistent across the length of the bioengineered construct. The basement membrane is lined with regularly spaced hemidesmosomes that anchor epithelial cells, indicating a robust adhesion between the compartments. Furthermore, evidence of caveolae at the basement membrane is indicative of active cellular communication and crosstalk between the epithelial and stromal compartments (Figure 6c,*).

Not only are epithelial cells anchored to the basement membrane by appropriate junctional complexes but also several intercellular junctions were observed via ultrastructural analysis. Electron dense bilaterally symmetrical desmosomes were located basally between adjacent cells (Figure 6d,*) and collections of junctions were located apically between neighbouring ciliated (Figure 6e) and goblet cells (Figure 6f) resembling tight junctions, adherens junctions and desmosomes consistent with apical junctional complexes ubiquitous within the human respiratory epithelium. The formation of such complexes is essential for a tissue to fulfil its protective role while enabling a level of permeability akin to its native tissue counterpart.

## 4 DISCUSSION

Bioengineered tissues have gained popularity as tools to enhance our understanding of biological processes, particularly within the context of pharmaceutical screening. In vitro technologies have allowed for the development of tissue constructs in 3D space, offering an alternative to traditional 2D flat monocultures, which are inherently simple and lack anatomical and physiological relevance. Moreover, such simplified models are often associated with discrepancies in expectations as drugs move into clinical trials. One reason for this unpredictability is the notion that cell and tissue structure underpins its functionality due to specialisation; therefore, cells forced into an artificial stressful 2D environment exhibit altered gene and protein expression due to the process of mechanotransduction (Allcock et al., 2023). For this reason, the development of tissues in a 3D culture setting has been an essential development as we strive to generate meaningful predictive outcomes in vitro.

The nasal mucosa has become an attractive tissue type within the field of bioengineering due to the wide range of applications it lends itself within the pharmaceutical industry. As the world experienced the COVID‐19 pandemic, the biotechnology sector raced to develop vaccines, which has led to an increased need for accurate and robust in vitro platforms capable of high confidence screening (Laffleur & Bauer, 2021). Minimally invasive systemic delivery of vaccines and drugs via the nasal mucosa is often a preferred route of administration during immunisation against respiratory pathogens such as coronaviruses and influenza, particularly via the use of nebulisers and the treatment of children. Consequently, this has led to demand for suitable in vitro platforms capable of preclinical screening and assessment of the interaction of vaccines with the human nasal respiratory mucosa (Hawksworth et al., 2020).

Two of the most popular current screening approaches include ex vivo porcine tissue, which is limited by lifespan and lacks species specificity (Glorieux et al., 2007; Ladel et al., 2019; Samson et al., 2012; Starbæk et al., 2022; Tulinski et al., 2013; Wadell et al., 1999), or immortalised cell lines. The RPMI 2650 cell line is considered an industry standard for nasal mucosal permeation studies; however, RPMI 2650 cells were derived from an anaplastic squamous cell carcinoma of the nasal septum, and thus, their ability to model healthy tissue is questionable (Hayflick & Moorhead, 1961; Wu et al., 1985). Furthermore, when RPMI 2650 cells are cultured in 3D, the resultant epithelium is frequently unpolarised, undifferentiated and multilayered, lacking the specialised and native structure that is essential for nasal function (Barlang et al., 2024; Gerber et al., 2022; Ladel et al., 2019; Martin et al., 2024; Merkle et al., 1998; Sibinovska et al., 2022). Many attempts at bioengineering a nasal construct rely on modelling of the epithelium compartment alone. Again, this is limited as epithelial–stromal signalling and communication is absent from such models and is known to be essential for normal tissue development, function and homeostasis. There are few nasal mucosal constructs that incorporate a supporting stromal element, and those that do rely on exogenous animal‐derived collagen hydrogels populated with fibroblasts (Wengst & Reichl, 2010). Such models are limited in their physiological relevance as human stromal tissue contains a complex network of ECM components and molecules, which combined with the inherent variability of animal‐derived products, creates tissue constructs that are not best suited for human nasal flux studies (Agu et al., 2001; Merkle et al., 1998; Ndongo Sonfack et al., 2024; Yankaskas et al., 1985).

The anatomy of the respiratory nasal mucosa features a heterogeneous cellular population with a multitude of specialisations specific to its secretory, absorptive and protective functions. These include motile cilia present on the surface of columnar cells, microvilli on the surface of goblet cells, mucus secretions and intercellular junctions, all of which are key factors that influence absorption and bioavailability of drugs, along with the transport of antigens through the epithelium (Birkhoff et al., 2009; Lochhead & Thorne, 2012; Parry et al., 2025; Su et al., 2023). Accordingly, a bioengineered construct capable of forecasting predictive outcomes must accurately express these key microanatomical features. In this study, we report the construction of a novel human nasal mucosal model and undertake in‐depth characterisation to demonstrate that the construct possesses these essential ultrastructural attributes.

It is notable that most bioengineered tissue described in the literature is characterised at the histological level and the ultrastructure of such models is rarely reported upon. This is despite the importance of determining its functionality and the widely accepted view of how structure is related to function (McInnes et al., 2007; Mercier et al., 2019). Here, we not only report the advancement of a nasal mucosal construct that combines both epithelial and stromal compartments representative of the native tissue, but we also describe its ultrastructural characterisation using both scanning and transmission electron microscopy.

The stromal foundation upon which the overlying epithelium was constructed relies upon the neosynthesis of a complex network of endogenous ECM secreted by human respiratory fibroblasts cultured in a 3D environment. This results in a completely humanised, tissue‐specific, complex ECM, rich in fibronectin and collagen, capable of providing biochemical cues, which along with physical and paracrine signalling, promotes communication between the stromal and epithelial compartments. This is an important feature that has been previously recognised as being essential to promote the differentiation and maturation of bioengineered tissue systems (Freer et al., 2024; Goncalves et al., 2022). Furthermore, the inclusion of a robust stromal compartment is integral in the formation of a trilayered basement membrane de novo, rich in anchoring hemidesmosomes and budding caveolae, suggestive of intercompartment communication (Stea & D'Alessio, 2025).

The structure and anatomy of the epithelium within the bioengineered nasal mucosal model compares favourably to native tissue at both histological and ultrastructural levels. The epithelium itself has self‐organised in a pseudostratified manner and consists of a heterogeneous cell population with ciliated columnar cells, basal cells and goblet cells all identifiable across the length of the tissue. In‐depth ultrastructural analysis revealed the presence and correct localisation of junctional complexes including electron‐dense desmosomes between basal cells and neighbouring cells, alongside tight junctions and adherens junctional complexes within the apical region between ciliated and goblet cells (Zhang et al., 2023).

The synergy between the varied cell types and the presence of junctional complexes is integral to the correct functionality and barrier formation within the tissue. Collectively these features provide confidence that the tissue construct compares favourably to the native nasal mucosa. Additionally, these morphological characteristics were observed in all samples examined, of which three models per experimental set up and three independent experiments were conducted. The epithelial morphological hallmarks were consistent across the length of the tissue construct, which supports the notion that not only is the microanatomy of the native tissue recapitulated accurately but also the tissue construct itself is both robust and reproducible.

The mucociliary clearance system serves as an important protective function of the nasal mucosa that traps and removes pathogens from the proximal airway. Features of the mucociliary clearance system identified in the bioengineered construct include a carpet of cilia lining the surface of the epithelium, as visible through SEM analysis. TEM analysis identified the typical axonemal structure inclusive of the 9 + 2 microtubule arrangement, which is indicative of motile cilia found in the respiratory tract (Lee et al., 2020; Pinto et al., 2020). Furthermore, we have observed the production of mucins from resident goblet cells within the tissue construct, which has been confirmed by biomarker expression, electron microscopy and histochemical identification. It is clear that not only are goblet cells present, but they are active and secretory.

The presence of both branched and unbranched microvilli coated in glycocalyx was identifiable using high magnification, in‐depth imaging, which has not been reported in other bioengineered tissues. Given that glycocalyx is a key signalling component and protective agent, its presence in vitro speaks to the physiological relevance of the system (Busuttil et al., 1977; du Preez et al., 2022; Fonseca et al., 2025; Jafek, 1983; Kesimer et al., 2013; Stonebraker et al., 2004).

This study describes an in‐depth analysis of the microanatomical characteristics of our bioengineered airway construct, which accurately recapitulates many features of the native tissue architecture. However, further detailed investigations as to the functionality of the construct are required to further strengthen its characterisation. For example, measurements of barrier function such as transepithelial electrical resistance, permeability studies, molecular characterisation at a gene expression level, response to pharmacological interventions and movement of cilia. Although structure and function are intrinsically related to one another, a thorough characterisation of the construct's functional properties combined with its robust and reproducible nature would allow for its widespread adoption for the multitude of applications that it is well suited, ranging from fundamental research to pharmacological screening trials.

Unlike many air–liquid interface model systems that engineer an epithelial compartment in isolation and therefore lack the complex interactions between the stromal and epithelial compartments (Barlang et al., 2024; Martin et al., 2024), the construct described within this study combines structural and dynamic elements of both tissue compartments. However, although our bioengineered construct boasts a level of ultrastructural characterisation that has yet to be described in other systems, it could benefit from inclusion of some of the functional elements made possible by other chamber‐based systems such as dynamic airflow, fluid flow or mechanical stimulation (Brooks et al., 2022; Koch et al., 2023; Walls et al., 2024). Combination of our robust and well‐characterised microanatomical architecture with high throughput and dynamic microenvironmental systems such as tissue‐on‐a‐chip or bioreactor‐based systems (Capuana et al., 2022; Hudock et al., 2025) could provide a unique platform that addresses many of the challenges associated with in vitro pharmacological screening. Particularly as these environmental elements are known to impact cell and tissue functionality including cell differentiation and barrier function.

Given the positive correlation between reliable predictive outcome and three‐dimensional cell culture, the value of this construct within the space of in vitro pharmacological testing is unprecedented. Although the presence of a polystyrene scaffold is not strictly physiological and may impact the stiffness of the cellular microenvironment, previous studies in our bioengineered intestinal construct have demonstrated that this does not impede the reliability of permeability studies (Freer et al., 2024) and the enhanced physiologically relevant anatomical structure of such constructs enhances their predictive value (Allcock et al., 2023).

## 5 CONCLUSIONS

In this study, we have described the development of a complex, bioengineered nasal mucosal construct capable of recapitulating aspects of native tissue microanatomy. We present this novel platform, having conducted an in‐depth ultrastructural analysis which is important in the characterisation of model systems, yet notably lacking in other studies. We consider this system to represent a significant advancement over these existing methodologies which are not as well characterised and their structure does not often compare favourably to the native human mucosa, thereby limiting their predictive value. We describe the presence of features of the mucociliary clearance system, a strong stromal foundation comprised of tissue‐specific fibroblasts and endogenous ECM, along with complex cellular junctions and a *t*ri‐laminar basement. Each of these anatomical features are essential tissue hallmarks that govern functionality in vivo. We present this model system as a valuable resource that could be applied to supporting research into fundamental cellular mechanisms and could serve as a platform technology capable of streamlining clinical trials through production of valuable and predictive preclinical data sets.

## AUTHOR CONTRIBUTIONS

NC, BD, DB and SP helped develop the initial concepts behind the study. NC, BD, SB, DB and SP managed the progression of the study and met regularly to review updates and plans. SB was largely responsible for the experimental elements, data acquisition and analysis. SB, KG and SP were responsible for data interpretation. KG and SP drafted the original article, and all co‐authors reviewed and approved the final manuscript. SP is the principal investigator, and KG and SP share corresponding authorship.

## FUNDING INFORMATION

Funding for this study was provided in part by REPROCELL Europe Limited and AstraZeneca via a formal agreement with Durham University.

## CONFLICT OF INTEREST STATEMENT

DB is an employee of REPROCELL Europe Limited. At the time of data generation, BD and NC were employees and may have been shareholders of AstraZeneca, the manufacturer of the Fluenz®/FluMist® intranasal live‐attenuated influenza vaccine. SP collaborates and acts as a technical consultant for REPROCELL Europe Limited. SB has no conflicts to declare.

## Supporting information


Table S1.

## Data Availability

The data that support the findings of this study are available from the corresponding author upon reasonable request.
