# Supplementary material for: Reconstruction of native cellular microanatomy in a novel bioengineered full thickness human nasal mucosal construct
Source: J Anat. 2026 Mar 16:10.1111/joa.70130. Online ahead of print. doi: 10.1111/joa.70130 (PMC13398369; doi:10.1111/joa.70130)
Supplement: Supplementary file 1 — Table S1. [file JOA-9999-0-s001.docx]

**Supplementary Table S1:** Antibodies used in immunofluorescence analysis

|  | **Target** | **Supplier** | **Product Code** | **Dilution** |
| --- | --- | --- | --- | --- |
| Primary Antibodies | Fibronectin | Abcam | Ab32419 | 1:100 |
|  | Collagen I | Abcam | Ab34710 | 1:100 |
|  | Integrin α6 | Abcam | Ab181551 | 1:100 |
|  | MUC5AC | Abcam | Ab3649 | 1:100 |
|  | Keratin-14 | Abcam | Ab7800 | 1:100 |
|  | Β-tubulin | Abcam | Ab315214 | 1:600 |
|  | E-cadherin | Abcam | Ab1416 | 1:100 |
|  | Ki67 | Abcam | Ab16667 | 1:100 |
|  | p63 | Abcam | Ab124762 |  |
| Secondary Antibodies | AlexaFlour 488 anti-mouse IgG | Abcam | A21202 | 1:1000 |
|  | AlexaFlour 594 anti-rabbit IgG | Abcam | A21207 | 1:1000 |
